# Scanning X-ray Fluorescence Data Analysis for the Identification of Byzantine Icons’ Materials, Techniques, and State of Preservation: A Case Study

**DOI:** 10.3390/jimaging8050147

**Published:** 2022-05-23

**Authors:** Theofanis Gerodimos, Anastasios Asvestas, Georgios P. Mastrotheodoros, Giannis Chantas, Ioannis Liougos, Aristidis Likas, Dimitrios F. Anagnostopoulos

**Affiliations:** 1 Department of Material Science and Engineering, University of Ioannina, 45110 Ioannina, Greece; fgerodim@uoi.gr (T.G.); a.asvestas@uoi.gr (A.A.); g.mastrotheodoros@inn.demokritos.gr (G.P.M.); 2 Conservation of Antiquities & Works of Art Department, West Attika University, 12243 Aegaleo, Greece; 3 Department of Computer Science and Engineering, University of Ioannina, 45110 Ioannina, Greece; gchantas@uoi.gr (G.C.); arly@cs.uoi.gr (A.L.); 4 Art Restoration E.E., 45500 Ioannina, Greece; liougosioan@gmail.com

**Keywords:** MA-XRF, elemental maps, clustering, dimensionality reduction, painting stratigraphy, pigments, panel painting

## Abstract

X-ray fluorescence (XRF) spectrometry has proven to be a core, non-destructive, analytical technique in cultural heritage studies mainly because of its non-invasive character and ability to rapidly reveal the elemental composition of the analyzed artifacts. Being able to penetrate deeper into matter than the visible light, X-rays allow further analysis that may eventually lead to the extraction of information that pertains to the substrate(s) of an artifact. The recently developed scanning macroscopic X-ray fluorescence method (MA-XRF) allows for the extraction of elemental distribution images. The present work aimed at comparing two different analysis methods for interpreting the large number of XRF spectra collected in the framework of MA-XRF analysis. The measured spectra were analyzed in two ways: a merely spectroscopic approach and an exploratory data analysis approach. The potentialities of the applied methods are showcased on a notable 18th-century Greek religious panel painting. The spectroscopic approach separately analyses each one of the measured spectra and leads to the construction of single-element spatial distribution images (element maps). The statistical data analysis approach leads to the grouping of all spectra into distinct clusters with common features, while afterward dimensionality reduction algorithms help reduce thousands of channels of XRF spectra in an easily perceived dataset of two-dimensional images. The two analytical approaches allow extracting detailed information about the pigments used and paint layer stratigraphy (i.e., painting technique) as well as restoration interventions/state of preservation.

## 1. Introduction

XRF spectrometry is extremely valuable in the field of cultural heritage investigation mainly because it offers rapid, accurate and non-invasive elemental characterization [1]. X-rays allow for a sophisticated analysis that can eventually lead to the extraction of information pertaining to the substrate(s) of an artifact. In addition, one can retrieve information on paintings’ materials and techniques through the generation of elemental distribution maps by scanning macroscopic X-ray fluorescence (MA-XRF), an approach that is being increasingly applied [2,3,4,5]. However, data acquisition during MA-XRF measurements results in a large number of spectra (often in the order of millions) and their analysis/visualization requires the development of efficient processing methods. Data analysis methods and strategies have been developed for processing MA-XRF data to create single element maps ([6,7] and reference therein); the latter allow extracting information about the employed pigments and painting technique and previous restoration interventions/state of preservation. Moreover, analysis of the intensity ratios of the characteristic transitions may provide information about the object’s stratigraphy [8,9].

However, spectral data are high dimensional; therefore, intelligent data analysis methods are needed to achieve data summarization and visualization in view of drawing conclusions about the existence of patterns and structure [10,11,12]. Here we have considered clustering methods to partition the data into groups containing spectra of similar shape. Spectra being of high dimensionality, we employed the well-known k-means clustering algorithm [13,14] that performs grouping based on the Euclidean distance between the spectrum vectors. The algorithm is simple, has low computational cost and provides sensible results. Once the clusters have been determined, representative spectra that summarize the spectral characteristics of each cluster can be computed. A notable difficulty when applying clustering methods is the determination of the correct number of clusters which is unknown in our case. To tackle this issue, we used a criterion called silhouette [15] that evaluates the quality of a clustering solution. We solved the clustering problem for several values of clusters’ number, evaluate each solution using silhouette and then selected the clustering solution of maximum silhouette value. Once the partition of spectra into clusters was obtained, a representative spectrum for each cluster was computed; the latter corresponds to the average of all spectra that belong to a given cluster and can be inspected to draw conclusions about the spectral characteristics of the cluster.

Another data analysis method we employed is dimensionality reduction; its aim is to map the original high-dimensional data into a low dimensional projection space under the condition that the relative distances among spectra are maintained in the projection space. Such a projection operation aims to eliminate irrelevant information and create low dimensional projections that convey most information included in the original data. Here, we first applied the well-known principal component analysis (PCA) approach (which is a linear projection method) to lower the dimension of the original data [16]. Subsequently, we applied the non-linear t-distributed stochastic neighbor embedding (t-SNE) method [17] to the PCA projected data to further reduce the data dimension to two. Once the two-dimensional projection vector of each spectrum was computed, it is possible to plot those data thus achieving spectra visualization [18].

**Figure 1:**
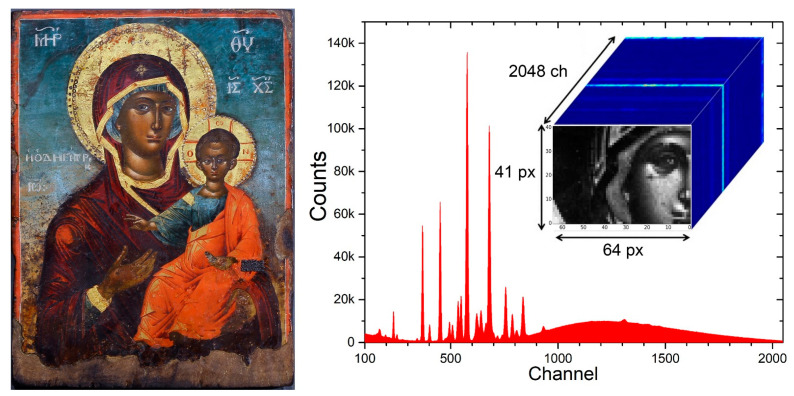
**Left:** Virgin Mary “Odigitria”, 18th century. **Right:** Sum of the 2665 XRF spectra acquired during the current scanning. Inset: the analyzed area. Every single spectrum and its (x, y) spatial position define a pixel.

The potentialities of our approach are explored through the examination of an 18th century Greek religious panel painting (“icon”) from Epirus (NW Greece) that depicts the Virgin Mary “Odigitria” (Hodēgētria = She who shows the Way) (Figure 1-left). The icon measures 28 × 21 cm^2^ and is attributed to the famous painters from Kapesovo village, the so-called “Kapesovites”. The Kapesovites were active during the 18th and 19th centuries and decorated tens of churches with wall paintings [19]. In addition, they were manufacturing portable icons, of which many dozens survive today [19,20]. Note that a growing interest in the investigation of icons’ materials and techniques has currently emerged, which is demonstrated by numerous publications [21,22,23,24,25,26,27,28]. In fact, the art of icon painting has been practiced in Greece since the dawn of Christianity and persisted even during the period between AD 1453 and 1830 (“post-Byzantine”) when the majority of Greek territories were under Ottoman Turk rule [29,30]. During the latter period, Greek painters remained largely adherent to the Byzantine tradition regarding painting style and kept using traditional painting materials and techniques [21,28]. For instance, despite the established use of oil mediums on primed canvas supports in Central European painting, Greek painters continued to employ egg yolk on gypsum grounds [31]. Moreover, they relied heavily on craftsmen’s handbooks for retrieving guidelines and prototypes (sketches/drawings) [32], a habit shared by most of the medieval painters in Europe [33]. Therefore, it is not surprising that the post-Byzantine icons were manufactured in a well-defined and rather conservative manner in terms of materials and techniques.

## 2. Materials and Methods

### 2.1. Experimental Setup

XRF scanning was performed using the M1-Mistral (Bruker) micro-XRF spectrometer which is equipped with a thick glass window (of ~2 mm thickness) microfocus X-ray W-tube, providing a continuous excitation spectrum emerging from 10 keV. Interchangeable beam collimators determine the beam spot on the target. The sample is positioned on a motorized X-Y-Z translation table. The X-Y stage allows programmable and remote measurements across the sample’s surface, either at specific points or as line and area scans. The travel range of the sample stage is about 180 mm in the x and y direction, with a minimum step size of 10 μm. The Z-stage controls the movement of the sample along the vertically impinging ionization beam, thus allowing the spectrometer’s geometrical arrangement to remain unchanged when targets with uneven surfaces are investigated. This is accomplished by the autofocus function, which is realized with the assistance of an optical microscope (magnification ×30) and four LED lights for sample illumination. The X-ray radiation emitted by the sample is detected by a semiconductor silicon drift detector (SDD) with an active silicon area of 30 mm^2^ and silicon thickness of 450 µm. The energy resolution is determined by the broadening (full width at half maximum, fwhm) of a Gaussian distribution describing the measured line–shape of single energy photons. The fwhm is a function of the impinging photon energy $E_{\varphi },$ and it is determined by the following equation:

(1) $$fwhm\left(eV\right)=\sqrt{2.47\cdot E_{\varphi }\left(eV\right)+4400}$$

Scanning was performed in a step-by-step mode. An area of 64 × 40 mm^2^ was scanned, with a lateral step of 1 mm in x- and y- directions. A circular-shaped aperture in the direction of the excitation beam provided a beam spot described by a two-dimensional Gaussian distribution, with spatial broadening of about 800 μm (fwhm). The X-ray tube working conditions were 800 µA, 50 kV, while the acquisition time was 2 sec for each point, thus resulting in about 1.5 h of active time for data collection. In Total, 2665 spectra were collected and stored, one for every measured point. Each of the raw spectra reflects the distribution of the measured photons over the 2048 channels. The measured (x, y) spatial position and the corresponding raw spectrum define a pixel. The total 2665 pixels are stored in a hierarchical data format (HDF) format file, creating the X-ray cube (Figure 1-right). In this way, the total information contained in the cube allows effortless data management. As an example, the summation of intensities per channel generates the sum intensity spectrum (Figure 1-right).

### 2.2. Clustering and Visualization

Our data analysis methodology is based on data clustering and visualization. Let $X=\left\{x_{1},x_{2},\ldots ,x_{N}\right\}$ denote the spectra dataset, where each x_i_ is a spectrum vector. The main objective of data clustering algorithms is to organize the spectral information by grouping similar spectra. More specifically, clustering methods partition the dataset *X* into groups (clusters) such that spectra in a given cluster are close in terms of a distance measure. Since spectra in the same cluster are similar, the information in each cluster can be summarized by a representative spectrum, the cluster centroid. By inspecting the properties of the centroid spectra, one can easily gain insight of the spectral information in the dataset. Moreover, in the case of spectral images, the spatial distribution of the clusters across the image can be visualized by plotting the ‘cluster labeled’ image, where pixels in the same cluster are plotted with the same color.

Since the spectra are high dimensional vectors, we have selected the most popular clustering method, namely the k-means algorithm [13,14,15] due to its simplicity and low computational complexity. The k-means divides the spectra dataset into k non-overlapping groups so that the variance of the clusters is minimized. It is an iterative algorithm that starts from a random initial clustering, uses the Euclidean distance as a proximity measure and at each iteration performs two steps to improve the partition. First, it assigns each spectrum to the cluster whose centroid is the closest and then recomputes the centroids of the new clusters. In this way, compact (of low between-cluster variance) cluster solutions are obtained. Since k-means depends on initialization, we executed the algorithm several times from different random initial partitions and finally kept the solution with the lowest between-cluster variance.

It should be emphasized that to apply k-means, the number of clusters K should be specified in advance. In our approach, this issue was tackled with the use of the silhouette criterion [15]. Silhouette is a measure used to evaluate the quality of a clustering solution ranging from −1 (highly overlapped clusters) to 1 (well separated clusters) [34]. Obviously, solutions with higher values are preferred. To use silhouette to select the number of clusters, k-means is executed for several values of the number of clusters K, each solution is evaluated using silhouette, and finally, the clustering solution of maximum silhouette value is selected. In our experiments, we considered solutions with more than six clusters and found that the k-means solution with K = 10 clusters provided the maximum silhouette score (≈0.52). Note that although less than seven clusters can achieve better scores, such a limited number of clusters does not allow for the analysis to be detailed. More specifically, a cluster number below seven would hinder the extraction of valuable information through the clustering process. In other words, for the present problem, we need more than seven clusters so the clustering results contain information with a level of detail suitable for our analysis objectives. For example, a cluster produced with K = 6, which corresponds to a specific part (color) of the painting, may consist of two or three parts (sub-clusters), each one highlighting the multi-layered structure of the painting. Thus, for, e.g., K > 7, the clustering output will contain these parts as concrete, separate clusters.

In addition to clustering, we also considered data visualization. For this reason, we applied dimensionality reduction methods to project each high-dimensional spectrum vector to a two-dimensional vector. Next, the two-dimensional projections were plotted, thus achieving spectra visualization.

As the spectrum vectors contain more than 2000 components, the principal component analysis (PCA) [35], a linear projection algorithm that produces the new independent features using linear combinations of the initial features, was first applied to reduce the dimensionality of the spectra to 25.

To further reduce the spectra dimensionality from 25 to 2, t-distributed stochastic neighbor embedding (t-SNE) was applied on dataset X’. The t-SNE is a nonlinear projection method, commonly used for mapping high-dimensional vectors x_i_ to low-dimensional vectors y_i_ to preserve the main structure of the initial data [35]. The t-SNE is generally considered superior; however, its computational complexity does not allow its application for high-dimensional data, since it becomes too slow in practice. On the other hand, even though PCA is a widely used technique, its major drawback is that it fails to maintain the local structures of the dataset. To mitigate this issue, a combination of PCA and t-SNE was herein selected. The data analysis was realized in Python with the use of Anaconda and Jupyter notebook environments.

## 3. Results

### 3.1. Data Analysis and Interpretation

Prior to commenting on the analytical data, we shall make a brief note regarding the technique of post-Byzantine icon painting. The painters of the post-Byzantine period were acting in a conservative technical framework that largely reflected the techniques of medieval European painting. Indeed, they worked mainly by mixing pigments with egg yolk (plus water), which is a medium that dries almost instantly, thus allowing for the rapid application of successive paint layers. This property of the medium affects the way an icon is painted. First, the preliminary underpaintings are applied. Then, they are partially covered by paint layers of successively lighter tonalities (midtones/highlights) [36]. This is extremely important and must be taken into account when interpreting XRF data because it implies that when analyzing an area where volume has been rendered (e.g., cheek), the XRF spectra may contain information that pertains to layers existing below the visible ones.

**Figure 2:**
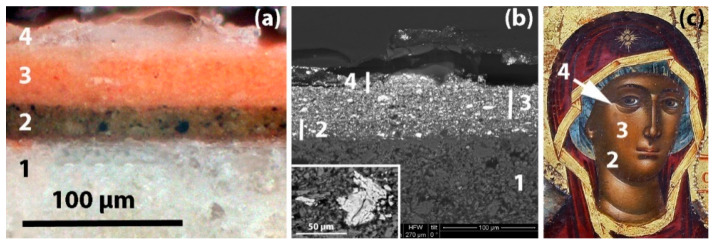
(**a**) Cross-section of a microsample; numbers mark the successive paint layers that lay on the ground (1); detail of an OM image (200×). (**b**) Same cross-section as seen through the SEM’s BSE detector (1000×). Note the extensive use of lead white grains (white particles) in all three flesh paint layers (2–4). (**c**) Detail of the Virgin Mary icon showing the flesh primary color (2), midtones (3) and highlights (4).

To demonstrate the typical structure of an icon, two photomicrographs of a characteristic micro-sample collected from a spot that depicts flesh is presented (Figure 2a,b); the first photomicrograph was captured through an optical microscope (OM) and the second using the backscattered electron detector (BSE) (the BSE detector allows for the differentiation of an observed phase on the basis of the atomic number of its constituent elements (the higher their atomic number, the brighter they appear) [37]) of a scanning electron microscope (SEM). This sample originates from an AD 1773 Greek icon that was painted by a Kapesovitis painter, such as the Virgin Mary icon in consideration. The sample’s cross section shows four distinct layers: the first (1) corresponds to the ground layer (“gesso”) on which the primary flesh color (2), the midtones (3) and the highlights (4) were applied. For reasons of convenience, a corresponding detail of the Virgin Mary icon with the various aforementioned paint layers marked on it is also shown (Figure 2).

### 3.2. Spectroscopic Analysis

The first method of analysis and interpretation of MA-XRF data is based on the extraction of element maps by analyzing each spectrum separately and then visualizing the extracted information in a two-dimensional spatial map. A calibration procedure is applied to X-ray cube spectra to convert each channel to photon energy. The applied calibration equation is given below:

(2) $$E_{\varphi }\left(eV\right)=19.99\left(eV/ch\right)\times channel-964.70\;eV$$

**Figure 3:**
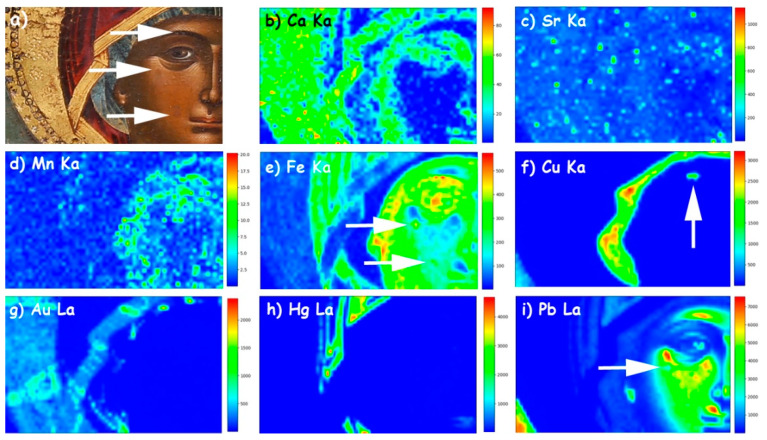
(**a**) Macro photograph of the scanned area; (**b**) Scanning XRF image of the Ca Kα transition intensity; (**c**) Sr Kα map; (**d**) Mn Kα map; (**e**) Fe Kα map; (**f**) Cu Kα map; (**g**) Au Lα map; (**h**) Hg Lα map; and (**i**) Pb Lα map. Arrows indicate areas of later interventions.

Each spectrum consists of 2048 channels and the measured energy range extends up to 40 keV. The data were analyzed using the PyMCA code [6]. The extracted elemental distribution maps of Ca, Sr, Mn, Fe, Cu, Au, Hg and Pb are shown in Figure 3.

Lead is detected on the areas of flesh and especially on the midtones and highlights (Figure 3i), thus revealing the employment of lead white, which was practically the only white pigment used in panel painting until the 19th century [38]; note that lead white was extensively mixed with other pigments to impart brightness or/and body (Figure 2c). Lead is also detected on the bluish areas, especially in the case of the pale-bluish background (lower left corner) and, faintly, on the Virgin Mary’s kerchief, where it was probably mixed with a copper-based blue pigment; besides, copper blue pigments were widely used by painters of the era and area in consideration [26].

**Figure 4:**
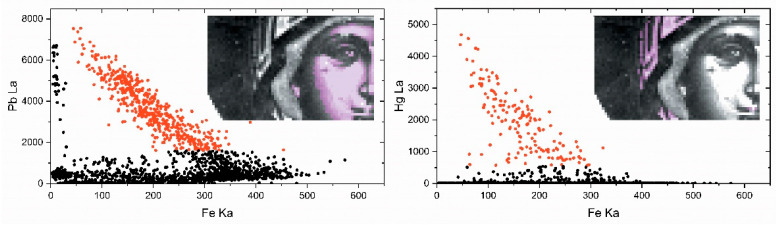
Transitions intensity scatter plots to derive spatial correlations. **Left**: Pb La relative to Fe Kα. Red scatter points correspond to measurement points over the Virgin’s highlighted part of the face. **Right**: Hg Lα relative to the Fe Kα intensity. Red scatter points correspond to measurement points over the highlighted part of the mantle.

Iron is detected on the flesh, on the mantle and, faintly, on the halo and the fringe of the mantle (Figure 3e). In flesh areas, there is a strong correlation between lead and iron. The intensities of the Fe Κα and the Pb Lα transitions are negatively correlated as can be deduced from their scatter plot (Figure 4-left). Here, iron is detected due to the employment of ochre-type pigments, which, in the case of flesh renderings, are mostly mixed in the primary paint layers (layer 2 in Figure 2a–c). Therefore, iron fades and gradually “disappears” in the areas of high lead content simply because the iron-rich primary layers lay below numerous layers of lead-rich paints (lighter tones and highlights—layers 3 and 4 in Figure 2a–c). However, in the areas of maximum iron intensities, there is also a strong presence of manganese (Mn) (Figure 3d), and this hints towards the employment of umber (i.e., a Mn-rich iron ochre) [28,39]. Moreover, these areas coincide with dark contours and shadows, where an umber pigment was presumably used.

The intensity of the Fe Kα transition on the mantle diminishes from the shadowy deep red areas towards the highlighted ones, where mercury (Hg) is also detected (Figure 3h). This is confirmed by the negative correlation between the Fe Κα and the Hg Lα intensities shown in Figure 4-right and indicates that the mantle highlights were rendered on top of the iron-ochre primary layer by cinnabar/vermilion (HgS), a pigment extensively used by post-Byzantine painters [28]. The same pigment was probably used for rendering the lips as indicated by the presence of mercury (Figure 3h). On the other hand, the minor iron that is detected on the gilded halo and the mantle fringe is obviously related to a “bole” substrate, namely, a fine, iron-rich clay that was customarily used for attaching gold leaf on icons [40]. The typical post-medieval gold leaf was extremely thin, (<1 μm) and this is probably why in the studied icon, low intensities of gold were detected (Figure 3g). However, post-Byzantine craftsmen had at their disposal finely ground gold powder too, and this material was often used like a pigment for rendering details on icons [40]. Interestingly, in the case of this artifact, the scanning XRF analysis indicates that both of these materials have been employed. The halo and mantle fringe appear as continuous areas, thus indicating employment of gold leaf, while scattered gold spots appear laying on the blue kerchief hinting towards the use of gold powder (“shell gold”) (Figure 3g). Note that the latter spots are of varying intensities, implying that the corresponding paint layers are of varying thickness. Moreover, the use of both gold leaf and powder is also indicated by the visual observation of the corresponding areas. It is worth mentioning that gold was extensively used in post-Byzantine painting to render icons’ backgrounds, saints’ halos and other iconographical details because this glittering and noble material was considered a representation of the divine glory [41].

Moreover, elemental maps reveal the presence of calcium and strontium (Figure 3b,c). Both these elements show greater intensities on areas that bear relatively thin layers, namely on the gildings (halo and fringe) and the areas of primary colors (darker/shadowy areas) of the face and mantle (Figure 3a), while their intensity drops drastically in the areas of high lead and high mercury intensities (e.g., flesh midtones and highlights). Therefore, it is inferred that calcium and strontium co-exist in a single layer that serves as a common substrate for both the paint layers and the gold leaf. This is indeed the case in the preparatory/ground layers (“gesso”) that were customarily applied on wooden panels prior to painting [31,42]. In the case of Greek post-Byzantine icons, grounds were almost exclusively made using calcium sulfate compounds (e.g., gypsum, anhydrite) [31], which often contain impurities of strontium sulfate (celestine—SrSO_4_) [31,43]. Interestingly, in the corresponding elemental map, there are several areas showing the intense presence of strontium, and this might indicate the presence of globules/particles. Previous SEM observations have revealed numerous strontium sulfate particles within the ground layers of post-Byzantine Greek icons (Figure 2b).

Finally, through the elemental maps, we were able to spot information pertaining to the state of preservation of the artifact. The lead elemental map shows two small-sized intensity irregularities on the area of Virgin Mary’s cheek, where lead is virtually absent presumably due to paint loss (arrows on Figure 3e), thus revealing older aesthetical interventions (“retouching”) performed using an iron-rich paint. Similarly, the elemental map of copper shows a spot of high intensity on the Virgin Mary’s eyebrow (Figure 3f). As the latter was originally rendered in umber (Figure 3d,e), the copper can be safely attributed to a later intervention. In all these cases, a close inspection of the icon reveals that these spots have indeed received retouching treatments to compensate for paint losses (Figure 3a).

### 3.3. Cluster Analysis

**Figure 5:**
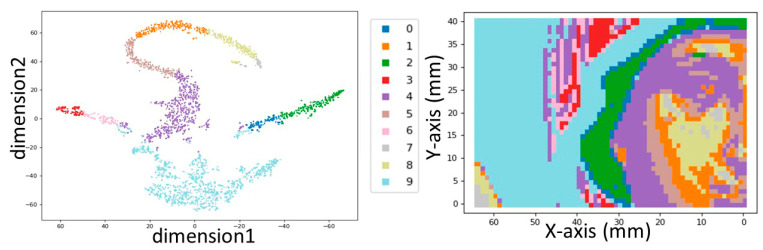
**Left**: Two−dimensional scatter plot of the 2665 measured points (pixels) distributed in 10 clusters, after dimensionality reduction. **Right**: Clusters distribution in the real space of the icon.

Our second approach for MA-XRF data interpretation is based firstly on the application of the k-means clustering algorithms to group the spectra with common features. This procedure groups the 2665 spectra contained in the X-ray cube spectrum to 10 distinct clusters. Then, principal component analysis (PCA) and t-distributed stochastic neighbor embedding (t-SNE) statistical methods were applied on the X-ray cube spectrum to allow for the visualization of the high-dimensional data in a two-dimensional scatter plot (Figure 5, Left). The spatial cluster distribution is shown in Figure 5, right, resembling the visual image.

**Figure 6:**
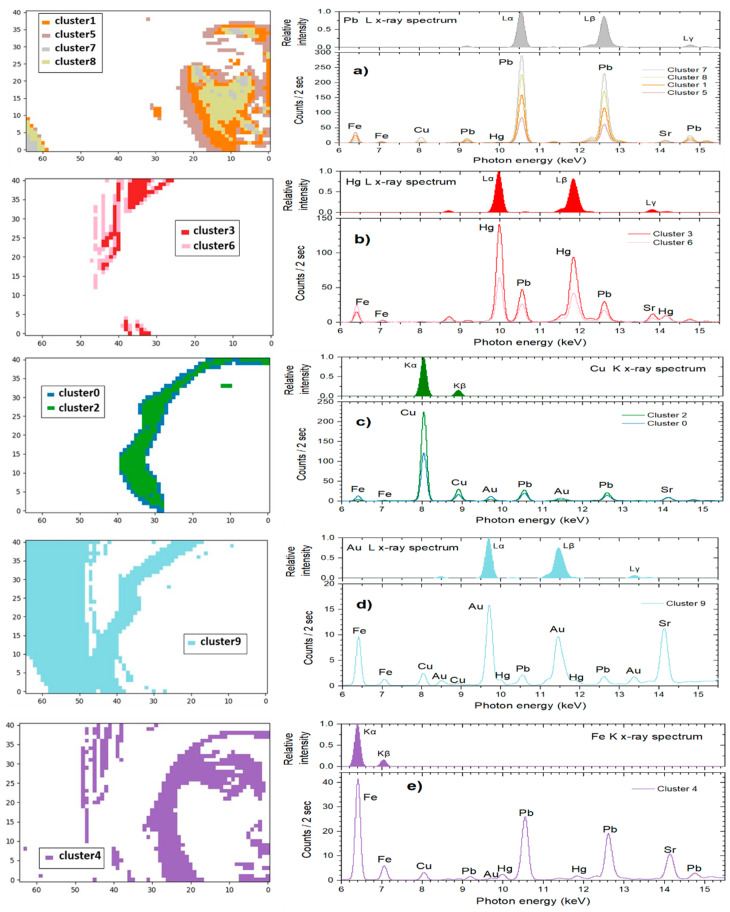
Spatial distribution of clusters (**left**); and corresponding X-ray emission spectra (**right**). On the top of the representative spectra is presented the X-ray spectrum of the dominating element, as evaluated by a Monte-Carlo simulation [44]. (**a**) Clusters 1, 5, 7 and 8. Pb Lα is the dominant transition. (**b**) Clusters 3 and 6; dominant transition: Hg Lα. (**c**) Clusters 0 and 2; dominant transition: Cu Kα. (**d**) Cluster 9; dominant transition: Au Lα. (**e**) Cluster 4; dominant transition: Fe Kα.

To extract information about pigments/materials, the representative spectrum of each cluster was evaluated. These spectra represent the mean spectrum of all spectra participating in each cluster. The correspondence between cluster distribution and the energy calibrated in the representative spectra (Equation (2)) is shown in Figure 6. Spectral lines analysis allows the determination of the elements involved in each cluster and, consequently, the presence of specific elements in the areas that each cluster describes. Moreover, the intensities of the transition lines are related to the mass concentration of the elements and their in-depth distribution, as well as the materials’ densities. The transition intensities were estimated by the region of interest method (ROI) which evaluates the intensity of each spectral line in an energy range defined between a low and a high energy limit, below and above the transition’s centroid, respectively. The energy range was selected equal to ±fwhm/2 (Equation (1)) to avoid overlap of the L X-ray transitions of the elements Au, Hg and Pb (with transition centroids at 9713, 9989 and 10,551 eV, respectively).

**Figure 7:**
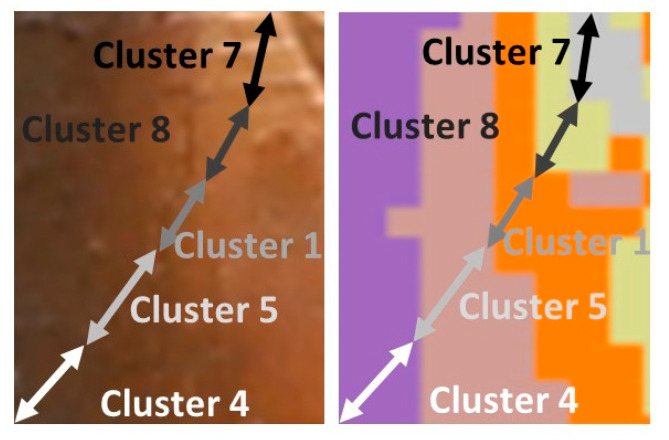
**Left**: Detail (macro photo) of Virgin Mary’s cheek where the multi-layered structure of the painting is evident. **Right**: Distribution of the various clusters that highlights the multi-layered structure of the painting.

**Figure 8:**
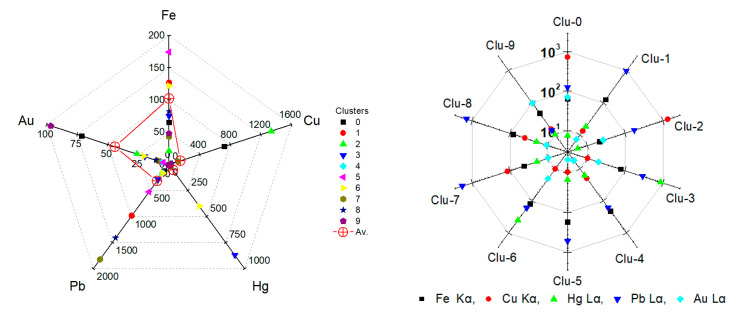
Intensities of Fe Kα, Cu Kα, Au Lα, Hg Lα and Pb Lα transitions for each cluster, extracted with the method of ROI from the representative spectra. **Left**: Intensities of specific transitions (that pertain to a specific element) for the various clusters. As an example, the maximum intensity of the Fe Kα is about 175 counts, and it is observed in Cluster 5, while the maximum intensity of the Cu Kα (~1300 counts) is in Cluster 2. The average transition intensities per pixel are marked by red crosses. **Right**: Transition intensities per specific cluster. For instance, in Cluster 2 the maximum intensity corresponds to the Cu Kα, followed by the Pb La.

The spatial distribution of Clusters 1, 5, 7 and 8, and the related representative XRF spectra are presented in Figure 6a. The group of clusters correspond to the image segment depicted in Figure 5, right, i.e., the Virgin’s flesh (face and ear) and the icon’s background (low left corner of the image). The Pb L X-ray spectrum dominates in the group, while the Fe K, Cu K and Sr K transitions are present. These four clusters that are grouped together contain the consistent characteristic transitions but with dissimilar intensities for each element. Especially in the case of the flesh, the distribution of the various clusters highlights the multi-layered structure of the painting and the fact that the whiter a pictorial element is, the more lead it contains (Figure 7). The extracted (by ROI method) intensities of the Fe Kα, Cu Kα, Au Lα, Hg Lα and Pb Lα transitions are shown in Figure 8. A negative correlation between lead and iron and a positive correlation between lead and copper is detected in Clusters 1, 5, 7 and 8. These correlations coincide with those revealed by the elemental maps analysis and indicate that a lead pigment was applied over an iron-rich pigment (flesh) and, in the second case, that the copper compound was mixed with the lead pigment (bluish background) (Figure 4). The intensity ratio Lβ/Lα of the Pb transitions was found to increase totally by 9% in the clusters sequence 5 → 1 → 8 → 7 (Figure 7, left), indicating that the lead layer becomes gradually thicker [45]. Similarly, the intensity ratio Kβ/Kα of the Fe transitions was found to increase totally by 25% in the clusters sequence 5 → 1 → 8 → 7. This is due to the smaller attenuation of the more energetic Fe Kβ photons (7.1 keV) compared to the Fe Kα photons (6.4 keV) from the overlaying lead layer [46]. The results above demonstrate that the proper analysis of the transitions intensities from the clusters’ representative spectra allows the stratigraphy determination [8].

The representative XRF spectra of Clusters 3 and 6 are shown in Figure 6b. This group of clusters corresponds to the Virgin Mary’s mantle; here, the Hg L X-ray spectrum dominates, while contributions from Fe K, Pb L and Sr K transitions are discrete. The extracted intensities of the Fe Kα, Cu Kα, Au Lα, Hg Lα and Pb Lα transitions are shown in Figure 8. A positive correlation between Hg and Pb intensities is deduced, which indicates the mixing of the pertinent compounds before their application. This finding is interesting because the two major inorganic red pigments of post-Byzantine painting, namely red lead (Pb_3_O_4_) and cinnabar (HgS), were indeed often intermixed [28]. Moreover, a negative correlation between Hg and Fe intensity values is observed, which suggests that the two elements were applied interchangeably (Hg over Fe), a conclusion extracted from the elemental maps analysis as well (Figure 4).

On the other hand, the energy distribution of Clusters 0 and 2 that correspond to the areas of the Virgin’s kerchief (2: kerchief interior, 0: borderline), and the corresponding representative XRF spectra are shown in Figure 6c. The extracted intensities of the Fe Kα, Cu Kα, Au Lα, Hg Lα and Pb Lα transitions are shown in Figure 6. Evidently, the Cu K X-ray spectrum dominates in both clusters, followed by Pb. This coincides with the conclusion extracted through the elemental maps analysis, which indicates that the Virgin Mary’s bluish kerchief was rendered by mixing a Cu-based blue pigment with Pb-white. The lower Cu intensity (which is accompanied by Fe and Au) in Cluster 0 is due to the elemental distribution in the borderline between the kerchief and its surroundings (iron-containing flesh and mantle, and the gold-based halo and fringe). In addition, the Au Lα transition has its second-highest intensity in cluster 0 (Figure 8). This is due, not only to the scarf’s borderline with the gold-based halo, but also to the usage of shell gold for painting parts of the scarf. What is highly interesting is the existence of a small number of pixels of Cluster 2 outside the Virgin’s kerchief (Figure 6c). These copper-dominated pixels are located on the Virgin’s eyebrow, and they are attributed to a later intervention as concluded from the elemental map analysis (Figure 3c). This observation reveals the high sensitivity of the cluster analysis.

The spatial distribution of Cluster 9 and its representative XRF spectrum is presented in Figure 6d. Cluster 9 corresponds to the Virgin’s halo, where Au L X-ray transition lines predominate. The simultaneous detection of Fe, Cu, Pb and Hg transition lines presumably pertains to the presence of these elements either at the halo’s borderline or to Au coating above these elements, or, occasionally, to their existence as substrates lying below Au (i.e., iron-based bole that was used as the binding material for the gold leaves). It is worth noting that Cluster 9 forms an extended and rarefied structure in the 2D cluster distribution (Figure 5, right). This is due to the one order of magnitude lower intensity of the Au Lα transition compared to the predominant transitions in the other clusters.

Finally, the spatial distribution of Cluster 4 and its representative XRF spectrum (Figure 6e) corresponds to the dark areas of the Virgin’s face and scarf. Fe K X-ray spectrum dominates this cluster, but the contribution of Pb L X-ray is also high. Moreover, Cluster 4 can be considered the continuation of Cluster 5, with the intensities of Pb and Fe inverted. In addition, weak transition intensities of Cu, Hg and Au are also recorded, obviously due to the extended borderline of the areal region of Cluster 4. Finally,, Cluster 4 occupies the center of the diagram and borders with all cluster groups.

## 4. Conclusions

The present work aimed at analyzing images of a painting through the processing of a large number of corresponding XRF spectra acquired through MA-XRF spectroscopy. Specifically, 2665 spectra from a segment of an 18th century post-Byzantine icon were recorded by a scanning spectrometer. To interpret the XRF spectra, two different methods were employed: a spectroscopic and a statistical data analysis one (“clustering approach”). The spectroscopic approach separately analyses each one of the measured spectra. This procedure requires fundamental knowledge of XRF spectroscopy, knowledge of the spectrometer’s energy calibration parameters and appropriate software for fitting theoretical models to the experimental data. The prominent advantage of the method is the construction of single-element spatial distributions images (element maps), with the spatial resolution determined from the scanning step and/or the X-ray beam spot.

The clustering approach was accomplished using a k-means algorithm. The advantage of the method is the grouping of thousands of XRF spectra in an easily perceived dataset of 10 clusters, without requiring distinct skills in XRF analysis and knowledge of energy calibration parameters. The obtained cluster distribution gave a fair description of the icon’s visual image. Subsequently, the representative spectra of each cluster were energy calibrated, thus revealing the strong correlation between clusters and elemental distribution. Further analysis was performed with dimensionality reduction algorithms. The aim of such a technique was to map the original high-dimensional data into a low-dimensional projection space under the condition that the relative distances among spectra are maintained in the projection space. While the extracted spatial resolution is inferior to the analysis by the elemental maps approach, the extracted maps are multi-elemental.

In conclusion, both of the applied analytical approaches allow for the extraction of detailed information about the employed pigments, the stratigraphy of the paint layers (painting technique) and the restoration state of the preservation of the artifact in consideration.

## Data Availability

Data is contained within the article.
